# Power and Coherence in the EEG of the Rat: Impact of Behavioral States, Cortical Area, Lateralization and Light/Dark Phases

**DOI:** 10.3390/clockssleep2040039

**Published:** 2020-12-09

**Authors:** Alejandra Mondino, Matías Cavelli, Joaquín González, Lucía Osorio, Santiago Castro-Zaballa, Alicia Costa, Giancarlo Vanini, Pablo Torterolo

**Affiliations:** 1 Laboratorio de Neurobiología del Sueño, Departamento de Fisiología, Facultad de Medicina, Universidad de la República, Av. Gral. Flores 2125, Montevideo 11800, Uruguay; a.monvero@gmail.com (A.M.); mat.cavelli@gmail.com (M.C.); joaqgonzar@gmail.com (J.G.); luos.mail@gmail.com (L.O.); sancaszab@gmail.com (S.C.-Z.); acosta@fmed.edu.uy (A.C.); 2 Department of Anesthesiology, University of Michigan, 7433 Medical Science Building 1, 1150 West Medical Center Drive, Ann Arbor, MI 48109-5615, USA; gvanini@umich.edu; 3 Department of Psychiatry, University of Wisconsin, 6001 Research Park Blvd, Madison, WI 53719, USA

**Keywords:** sleep, REM, slow waves, oscillations, gamma, spindles

## Abstract

The sleep-wake cycle is constituted by three behavioral states: wakefulness (W), non-REM (NREM) and REM sleep. These states are associated with drastic changes in cognitive capacities, mostly determined by the function of the thalamo-cortical system, whose activity can be examined by means of intra-cranial electroencephalogram (iEEG). With the purpose to study in depth the basal activity of the iEEG in adult rats, we analyzed the spectral power and coherence of the iEEG during W and sleep in the paleocortex (olfactory bulb), and in neocortical areas. We also analyzed the laterality of the signals, as well as the influence of the light and dark phases. We found that the iEEG power and coherence of the whole spectrum were largely affected by behavioral states and highly dependent on the cortical areas recorded. We also determined that there are night/day differences in power and coherence during sleep, but not in W. Finally, we observed that, during REM sleep, intra-hemispheric coherence differs between right and left hemispheres. We conclude that the iEEG dynamics are highly dependent on the cortical area and behavioral states. Moreover, there are light/dark phases disparities in the iEEG during sleep, and intra-hemispheric connectivity differs between both hemispheres during REM sleep.

## 1. Introduction

The brain is a complex system, in which parallel processing coexists with serial operations within highly interconnected networks, but without a single coordinating center. This organ integrates neural events that occur at different times and locations into a unified perceptual experience [1,2]. Cognitive states are mostly determined by the function of the thalamo-cortical system [3]. Part of this neuronal processing can be accurately measured by intra-cranial electroencephalogram (iEEG) or electro-corticogram, which reduces artifacts from non-brain electrical activity, such as eye movement and muscle or electrode noise, and increases the quality of high frequency oscillations in comparison to standard surface electroencephalographic recordings (EEG) [4,5].

The sleep–wake cycle is a critical physiological process and one of the most preserved biological rhythms through evolution [6]. This cycle is composed of wakefulness (W), non-rapid eye movement (NREM) and rapid eye movement (REM) sleep states, that are distinguished by their behavior and electrophysiological signatures, which can be captured by iEEG signals [3,7]. Accompanying these electro-cortical differences among states, the cognitive capacities drastically change during the cycle. Fundamentally, consciousness is lost during deep NREM sleep, and emerges in an altered fashion during REM sleep, when most vivid dreams occur [3,8].

One of the most-studied neural correlates of consciousness are the cortical EEG oscillations [9], which contain broad and complex frequency spectra that can be examined by means of the fast Fourier transform. The power of the different frequencies of these signals reflect the local degree of synchronization of the extracellular potential, which are deeply modified on passing from W to sleep [10,11]. The power of the frequency components of the iEEG is deeply modified on passing from W to sleep. While W and REM sleep contain high frequency activity together with theta waves (5–9 Hz) in the iEEG, during NREM sleep oscillations with slower frequencies (delta band, 0.5 to 4 Hz) and spindles (sigma band, 11 to 15 Hz) predominate [3,12,13,14].

Synchronization between oscillations from different areas represent another neural correlate of consciousness [15]. In this regard, the degree of iEEG coherence between two cortical regions reflects the strength of the functional interconnections (re-entries) that occur between them [16]. In other words, the spectral coherence analysis of the iEEG is a valid approach to infer cortical connectivity and communication between distant brain areas [17]. Siegel et al. (2012) proposed that frequency-specific correlated oscillations in distributed cortical networks provide indices or “fingerprints”, of the network interactions that underlie cognitive processes [18]. During W, there is a larger coherence in gamma (35–100 Hz) and high frequency oscillations (HFO, up to 200 Hz) than during sleep [11,19,20,21]. A high degree of delta and sigma synchronization occurs during NREM sleep [22], while theta coherence is large during REM sleep in the rodent iEEG [21].

Although there are several studies that have analyzed the iEEG during W and sleep, most of them have not evaluated the whole spectrum of frequencies (from delta to HFO), the differences between brain areas, lateralization and the phases of the circadian cycle. Additionally, the ones that did, have focused in just one of these parameters without evaluating the effect of all of them [20,21,23,24,25]. Therefore, a detailed and systematic evaluation, that evaluates the impact of behavioral states, cortical area, lateralization and light/dark phases of power and coherence during behavioral states is still pending. Hence, the purpose of this study was to provide an examination of power and coherence of the basal iEEG activity of the adult rat during W and sleep. To this end, we studied the influence of the cortical site, employing electrodes located on the olfactory bulb (OB), frontal (primary motor or M1), parietal (primary somatosensory, S1) and occipital (secondary visual, V2) cortex, as well as the impact of laterality (differences in signals recorded in the right and left hemispheres) and the influence of dark and light phases. Upon considering this set of factors, we found important patterns of activity characterizing each sleep state along with state independent modulations of iEEG activity.

## 2. Results

**Figure 1:**
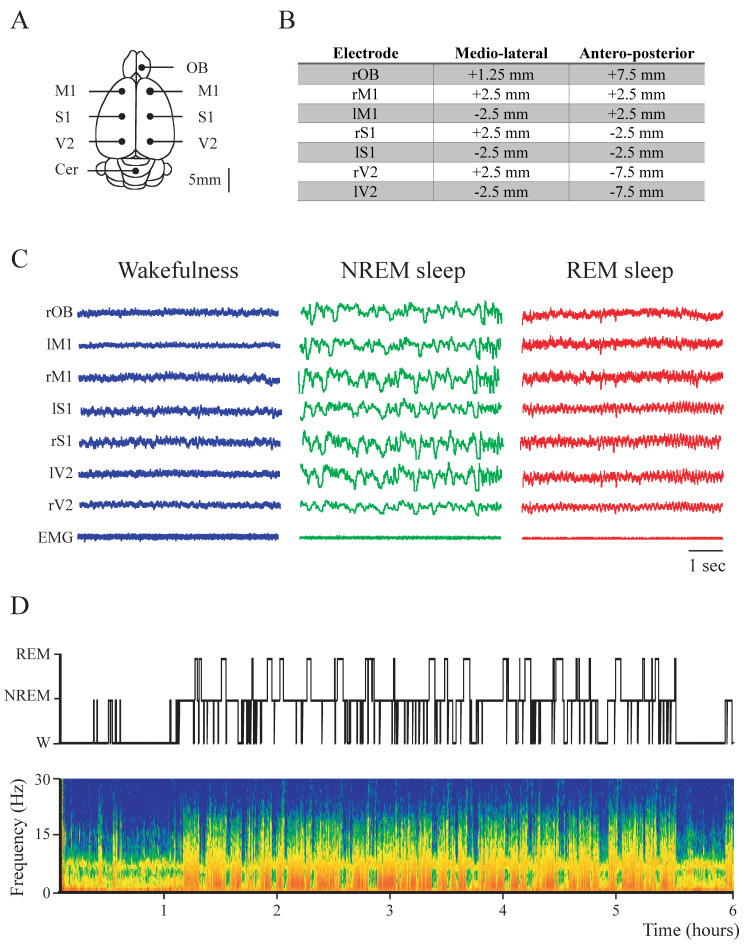
Sleep–wake states in the rat. (**A**) Schematic representation of the electrode position in the brain of the rat. (**B**) Electrodes’ positions in reference to *Bregma* [26]. OB, olfactory bulb; M1, primary motor cortex; S1, primary somato-sensory cortex; V2, secondary visual cortex; r, right; l, left. (**C**) Representative iEEG and the neck electromyogram (EMG) recordings during wakefulness (W, blue), NREM (green), and REM sleep (red). From top to bottom, olfactory bulb (OBr), right and left primary motor (M1r/M1l), primary somatosensory (S1r/S1l), and secondary visual (V2r/V2l) cortices. (**D**) Hypnogram (top) according to visually scored behavioral states and spectrogram (0.1 to 30 Hz). During W and REM sleep, theta activity (5–9 Hz) in the spectrogram can be readily observed. During NREM sleep, delta activity (0.5 to 4 Hz) is more prominent and there are intermittent episodes of sigma activity (10–15 Hz) which corresponds to the presence of sleep spindles.

Polysomnographic recordings, hypnogram and spectrogram (power spectrum as a function of time) during the light phase of a representative rat are displayed in Figure 1C,D. As it is exhibited in this figure, the quality of the recordings allowed for an optimal classification of W and sleep epochs.

### 2.1. Power Spectrum: Effect of Behavioral States and Recording Site

**Figure 2:**
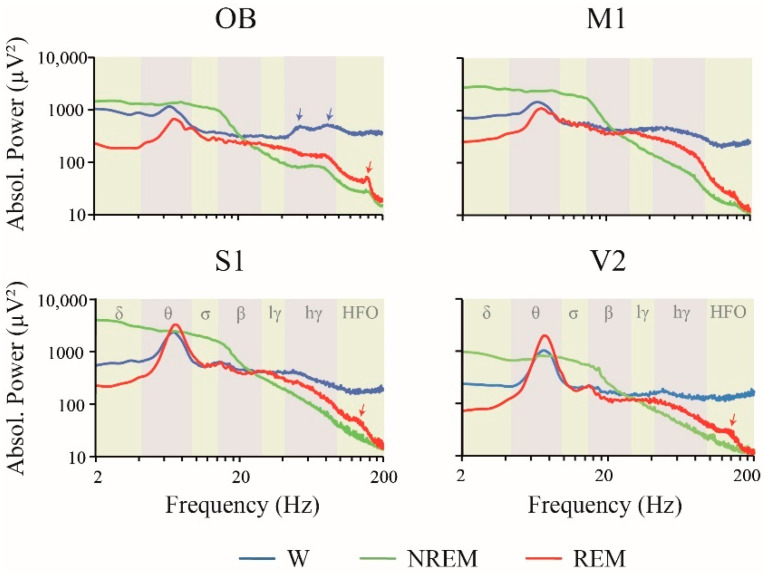
Power spectral profiles. Mean absolute power spectral profiles of the right hemisphere in wakefulness (W), NREM and REM sleep during the light period (*n* = 11). The frequency bands used for the statistical analysis are indicated by different colors in the background of the graphics. Blue arrows show two small deflections in the HG band during W and red arrows demonstrate a narrow peak in HFO at ≈130 Hz that can be appreciated during REM sleep in OB and somatosensory cortices. OB, olfactory bulb; M1, primary motor cortex; S1, primary somato-sensory cortex; V2, secondary visual cortex; W, wakefulness; r, right; l, left; lγ, low gamma or LG; hγ, high gamma or HG; HFO, high frequency oscillations.

Figure 2 shows the absolute power spectra analysis of the iEEG during W and sleep for the OB and neocortical areas during the light (resting) phase for the right hemisphere. In order to improve the visualization of the power differences among states, in the figure we multiplied the power at each frequency by the frequency itself. It is readily observed that the spectrum is highly variable in function of behavioral states and electrode sites.

The absolute power of all the frequency bands of the iEEG was affected by behavioral states, localization of electrodes and the interaction of both factors (Table 1). The exceptions were sigma, beta and low gamma (LG) that were not significantly affected by the interactions between behavioral states and electrode locations.

**Figure 3:**
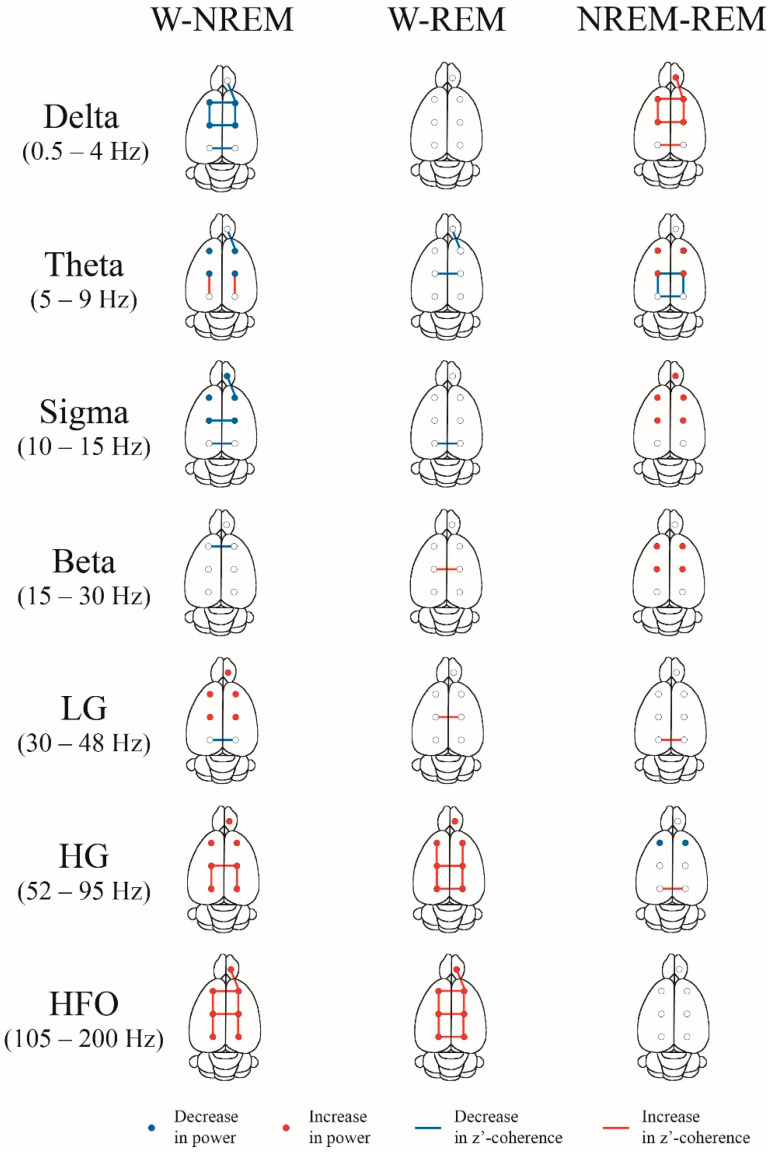
Summary of the power and z’-coherence. Statistically significant differences in absolute power and z’-coherence during wakefulness (W), NREM and REM sleep during the light phase. The circles represent the power for the different electrodes’ positions, while the lines represent the coherence for the different derivations. The results were evaluated by means of repeated measures mixed-effects model and Sidak test for multiple comparisons (*n* = 11). Blue represents a significantly (*p* < 0.05) lower difference between two behavioral states, and red a significantly higher difference. Power data are from the right hemisphere but are represented as bilateral. The complete statistics of these data are shown in Supplementary Figures S1 and S8.

A summary of the power spectrum differences between W, NREM and REM sleep is shown in Figure 3 (statistics are shown in Supplementary Figure S1A). The most remarkable results are the following. Delta, theta and sigma power during NREM sleep were significantly higher than during W and REM sleep in M1 and S1. In the OB, delta was larger during NREM compared to REM sleep, while sigma was larger during NREM compared to the other states. LG, HG and HFO powers were higher during W than during NREM in all the cortical areas. Additionally, HG and HFO, during W, was higher in comparison to REM sleep in most cortical areas. Finally, HG and LG power was also larger in REM sleep than in NREM sleep in M1, while in S1 this fact was observed only for LG.

**Figure 4:**
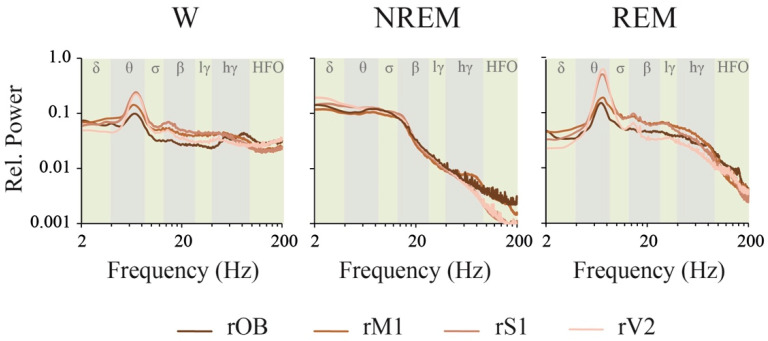
Relative power. Mean relative power profile of each behavioral state of the right hemisphere during the light period (*n* = 11). This approach removes the effect of the distance between the active and the referential electrode. The analyzed frequency bands are indicated by different colors in the background of the graphics. OB, olfactory bulb; M1, primary motor cortex; S1, primary somato-sensory cortex; V2, secondary visual cortex; W, wakefulness; r, right; l, left; lγ, low gamma or LG; hγ, high gamma or HG; HFO, high frequency oscillations.

In Supplementary Figure S2, the same tracings of Figure 2 were re-plotted for a more precise comparison as a function of the electrode location; the statistics of these data are shown in Supplementary Figure S1B. However, it is important to consider that the variation in the absolute power in the function of the electrode site is dependent of the distance between the active and the referential electrode (cerebellum). In this regard, as shown in Supplementary Figure S3, the total power was highly modified as a function of the electrode site (total power was also affected by behavioral states and the interaction with electrode localization). Indeed, total power was the lowest in V2 (closer to the reference electrode) reaching significance during W and NREM sleep. Then, we judged it to be more adequate to explore the relative instead of the absolute power in the function of the electrode site (nevertheless, complete statistics of the absolute power in the function of the electrode site are also provided in Supplementary Figure S1B). The analysis of the relative power in the function of the electrode site is exhibited in Figure 4 and Table 2 and Table 3 (the analysis of the relative power in the function of behavioral states is shown in Supplementary Figure S4).

The most remarkable differences in the relative power between the brain regions were noticed during REM sleep, where V2 and S1 theta power was greater than in OB and M1. Another interesting finding was that LG and HG in M1 were higher than in V2. During NREM sleep, S1 power was higher than OB for the delta frequency band, and larger than OB and M1 for the theta band. On the other hand, the power in the OB was lower than M1 and S1 for LG, but became higher for HG and HFO during W.

### 2.2. Power Spectrum: Light vs. Dark Phases

**Figure 5:**
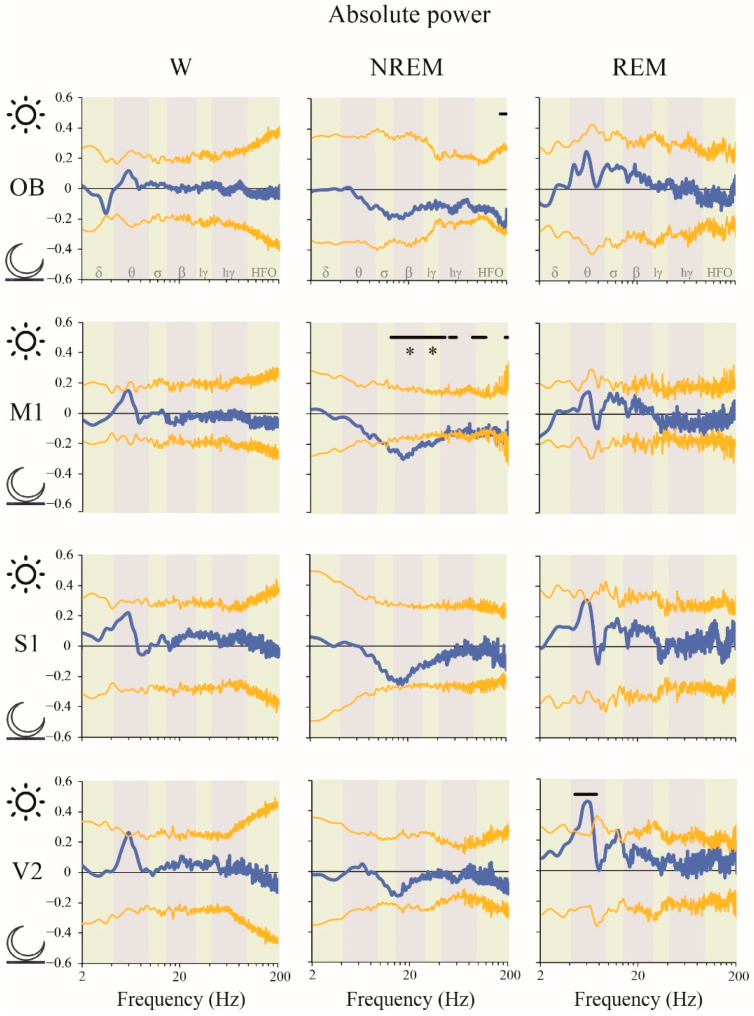
Absolute power: light vs. dark phase differences. The blue traces indicate the mean power difference between light and dark phases. The yellow lines represent the standard deviation of the mean with respect to zero. The statistical evaluation was performed by the two-tailed paired *t*-test with Bonferroni correction for multiple comparisons; * indicates significant differences, *p* < 0.0071 (*n* = 11). We also performed a data-driven approach comparing empirical clusters of frequencies; black lines represent statistical differences in cluster of frequencies, *p* < 0.05. In M1 the following frequency clusters were significantly larger during NREM sleep in the dark phase: 13 to 46 Hz (*p* = 0.001), 51 to 60.5 Hz (*p* = 0.016), 87 to 92 Hz (*p* = 0.038), 93.5 to 100 Hz (*p* = 0.025), 101 to 111 Hz (*p* = 0.012), 112.5 to 119.5 Hz (*p* = 0.024), 124 to 125.5 Hz (*p* = 0.047), 186.5 to 192 Hz (*p* = 0.036) and 193 to 200 Hz (*p* = 0.029). In the OB during NREM sleep, the power of the frequencies 171.5 to 187.5 and 188.5 to 199.5 Hz were larger during the night (*p* = 0.018 and *p* = 0.047, respectively). During REM sleep, the cluster 4.5 to 7.5 Hz was higher during the day than during the night in V2 (*p* = 0.038). The analysis was performed for the right hemisphere. OB, olfactory bulb; M1, primary motor cortex; S1, primary somato-sensory cortex; V2, secondary visual cortex; lγ, low gamma or LG; hγ, high gamma or HG; HFO, high frequency oscillations.

Next, we examined the effects of the light/dark phases on the iEEG oscillatory activity for the right hemisphere. To simplify, we only show the significant results, while the non-significant effects are shown in the Supplementary Material. Figure 5 shows the light/dark predominance (see the procedure in the figure legend; the same approach was used to show the data in the following figures). We can readily observe that the classic frequency bands show significant differences only during NREM sleep; beta and LG power were larger during the dark than during the light phase in M1. Employing a more precise evaluation using the empirical cluster analysis, we determined that during NREM sleep in M1 clusters of frequencies that include sigma, beta, LG, HG and HFO bands were larger in the dark phase. A cluster of frequencies within the HFO band also increased during the dark phase in the OB during this behavioral state. Regarding REM sleep, although no changes were found in the classical frequency bands, we found a specific cluster within theta frequency band which was higher during the day than during the night in V2.

### 2.3. Power Spectrum: Right vs. Left Hemispheres

iEEG absolute power laterality was analyzed for both the light (Supplementary Figure S5) and dark (Supplementary Figure S6) phases. Neither *t*-test evaluation of classical frequency bands nor cluster analysis showed statistical differences between right and left hemispheres, either during W or sleep.

### 2.4. Coherence: Effects of Behavioral States and Derivations

**Figure 6:**
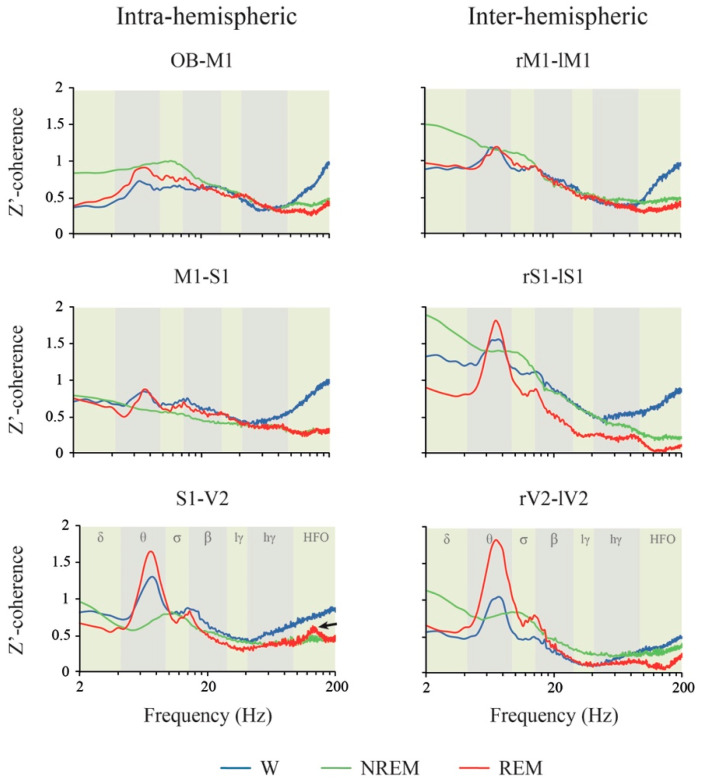
Z’-coherence. Mean z’-coherence profile of the inter-hemispheric and intra-hemispheric derivations (between adjacent areas) during wakefulness (W), NREM and REM sleep in the light phase (*n* = 11). The analyzed frequency bands are indicated by different colors in the background of the graphics. OB, olfactory bulb; M1, primary motor cortex; S1, primary somato-sensory cortex; V2, secondary visual cortex; r, right; l, left; lγ, low gamma or LG; hγ, high gamma or HG; HFO, high frequency oscillations.

The z’-coherence during W, NREM and REM sleep for the right intra-hemispheric and the inter-hemispheric combination of adjacent electrodes during the light period is shown in Figure 6. In Supplementary Figure S7, the data were re-plotted in order to appreciate the differences in function of the derivations. The statistical results of the repeated measures mixed-effects model are shown in Table 4. Interestingly, no significant differences in z’-coherence were observed in the function of the derivation, except for delta and theta bands. However, behavioral states and the interaction between localization and behavioral states modified z’-coherence in all the frequency bands (Table 4).

The spectral z’-coherence of each of the frequency band differences between W, NREM and REM sleep for each electrode combination is also shown in Figure 3; the p values for each comparison are shown in Supplementary Figure S8. The most important results are the following. Delta z’-coherence during NREM sleep was larger than during the other behavioral states in most derivations. Moreover, sigma z’-coherence had higher values during NREM than W in the intra-hemispheric combination OB-M1 and the inter-hemispheric motor and somatosensory cortices. Theta z’-coherence increases during REM sleep in comparison to NREM sleep in the posterior intra and inter-hemispheric combination of electrodes. HG and HFO intra and inter-hemispheric z’-coherence was larger during W compared to REM and NREM sleep in most derivations. Additionally, visual inter-hemispheric LG and HG z’-coherence were lower during REM sleep compared to NREM sleep.

**Figure 7:**
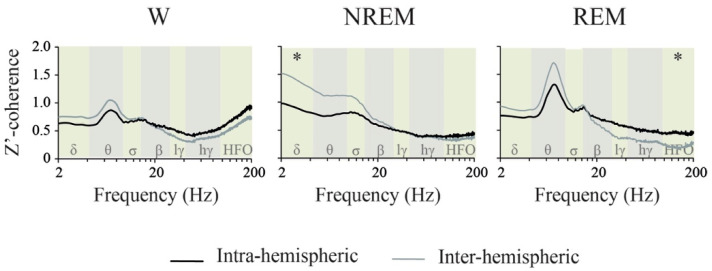
Z’-coherence differences between intra- and inter-hemispheric derivations. z’-coherence profile of the mean intra-hemispheric (OB-M1, M1-S1 and S1-V2) and inter-hemispheric (right–left M1, S1, V2) derivations during wakefulness (W), NREM and REM sleep in the light phase (*n* = 11). The analyzed frequency bands are indicated by different colors in the background of the graphics. OB, olfactory bulb; M1, primary motor cortex; S1, primary somato-sensory cortex; V2, secondary visual cortex; r, right; l, left; lγ, low gamma or LG; hγ, high gamma or HG; HFO, high frequency oscillations. Asterisks indicate significant differences, *p* < 0.05. M1, primary motor cortex; S1, primary somato-sensory cortex; V2, secondary visual cortex; lγ, low gamma or LG; hγ, high gamma or HG; HFO, high frequency oscillations.

When comparing z’-coherence in the function of the derivation, we could detect very few differences during W and sleep, and in most of the cases, this included differences between intra and inter-hemispheric derivations (Supplementary Figure S9). Therefore, we analyzed the differences between the average inter-hemispheric (right and left M1, S1 and V2 derivations) and intra-hemispheric z’-coherences (OB-M1, M1-S1 and S1-V2 derivations) during W and sleep (Figure 7). We found a significant effect of the interaction between the behavioral state and the derivation type for delta (F _(2,14)_ = 4.7, *p* = 0.028, η_p_^2^ = 0.402) and HFO bands (F _(2,14)_ = 3.8, *p* = 0.048, η_p_^2^ = 0.351). During NREM sleep, delta inter-hemispheric was higher than the intra-hemispheric z’-coherence (*p* = 0.046). In contrast, during REM sleep, HFO intra-hemispheric was larger than the inter-hemispheric z’-coherence (*p* = 0.038).

### 2.5. Coherence: Light vs. Dark Phases

**Figure 8:**
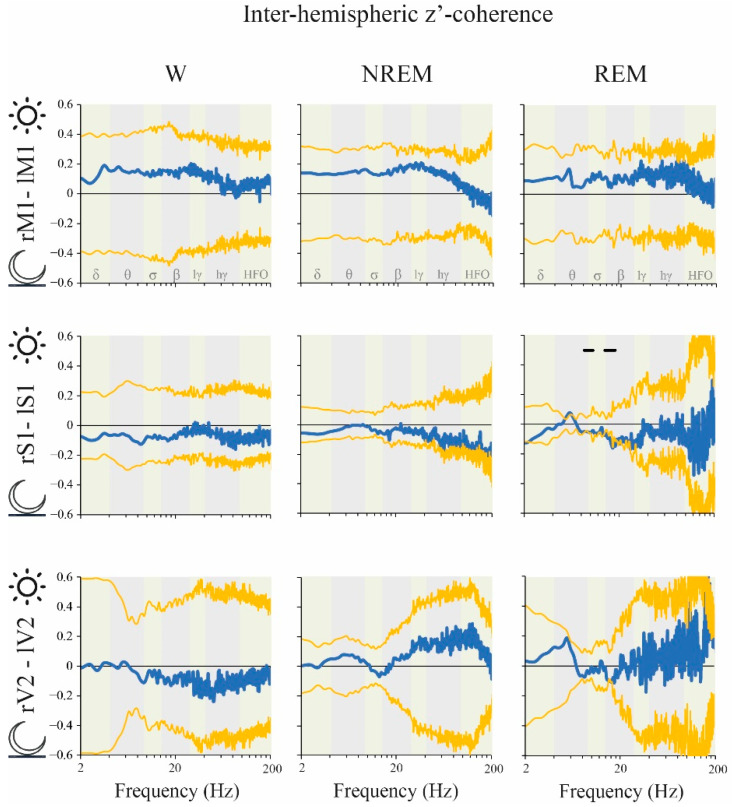
Inter-hemispheric z’-coherence: light vs. dark phases differences. (*n* = 11). The blue traces indicate the mean coherence difference between light and dark phases. The yellow lines represent the standard deviation of the mean with respect to zero. The statistical evaluation was performed by the two-tailed paired *t*-test with Bonferroni correction for multiple comparisons; no significant differences were observed. We also performed a data-driven approach comparing empirical clusters of frequencies; black lines represent statistical differences in cluster of frequencies, *p* < 0.05. During REM sleep, inter-hemispheric S1 z’-coherence was higher during the dark phase for the clusters 8.5 to 10.5 Hz (*p* = 0.008) and 14 to 18.5 Hz (*p* = 0.005). M1, primary motor cortex; S1, primary somato-sensory cortex; V2, secondary visual cortex; r, right; l, left; lγ, low gamma or LG; hγ, high gamma or HG; HFO, high frequency oscillations.

**Figure 9:**
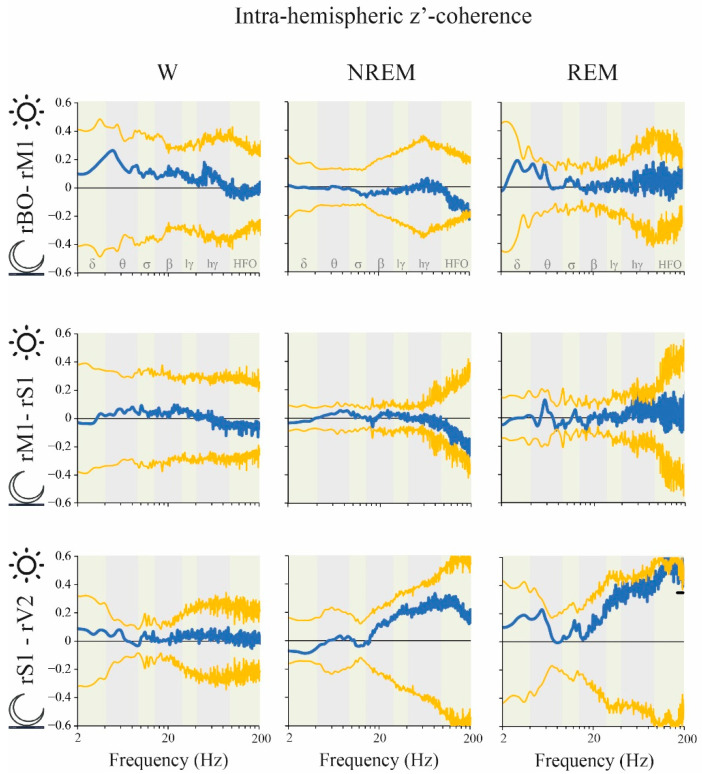
Intra-hemispheric z’-coherence: light vs. dark phases differences. The blue traces indicate the mean z’-coherence difference between light and dark phases. The yellow lines represent the standard deviation of the mean with respect to zero. The statistical evaluation was performed by the two-tailed paired *t*-test with Bonferroni correction for multiple comparisons; no significant differences were observed (*n* = 11). We also performed a data-driven approach comparing empirical clusters of frequencies; black lines represent statistical differences in cluster of frequencies, *p* < 0.05. During REM sleep, intra-hemispheric S1-V2 z’-coherence was higher during the light phase for the cluster 173–200 Hz (*p* = 0.005). OB, olfactory bulb; M1, primary motor cortex; S1, primary somato-sensory cortex; V2, secondary visual cortex; r, right; lγ, low gamma or LG; hγ, high gamma or HG; HFO, high frequency oscillations.

The influence of light/dark phases on z’-coherence was also analyzed. There were no significant differences for the classical frequency bands either for inter (Figure 8) or intra-hemispheric (Figure 9) z’-coherence between the dark and light phases during either W or NREM sleep. However, during REM sleep, inter-hemispheric S1 z’-coherence was higher in the dark phase for two clusters of frequencies: 8.5 to 10.5 Hz and 14 to 18.5 Hz (Figure 8). Moreover, REM sleep intra-hemispheric S1-V2 z’-coherence was higher during the light phase for the cluster 173–200 Hz (Figure 9).

### 2.6. Coherence: Right vs. Left Hemispheres

Neither *t*-test analysis of classical bands nor cluster analysis showed intra-hemispheric z’-coherence differences between right and left hemispheres during the light phase (Supplementary Figure S11).

**Figure 10:**
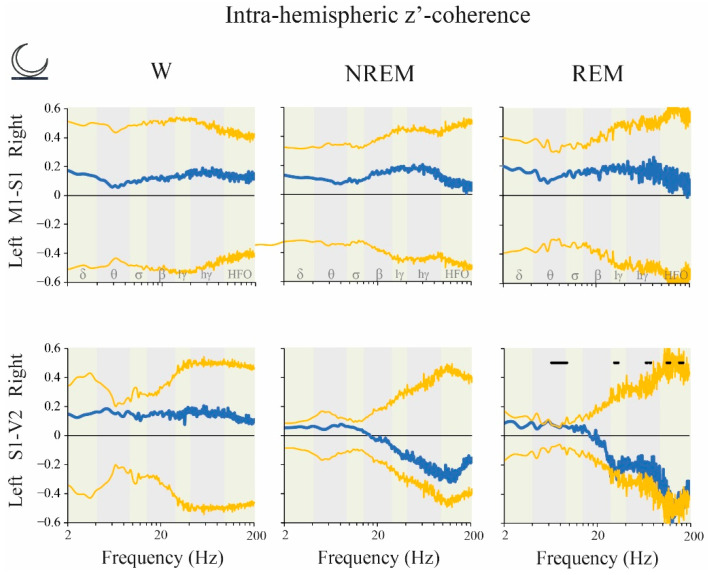
Intra-hemispheric z’-coherence: right vs. left hemispheric difference during the dark phase. The blue traces indicate the mean z’-coherence difference between light and dark phases. The yellow lines represent the standard deviation of the mean with respect to zero. The statistical evaluation was performed by the two-tailed paired *t*-test with Bonferroni correction for multiple comparisons; no significant differences were observed (*n* = 11). We also performed a data-driven approach comparing empirical clusters of frequencies; black lines represent statistical differences in cluster of frequencies, *p* < 0.05. Coherence was higher in the right hemisphere for the cluster between 6.5 to 9.5 Hz (*p* = 0.0009) during REM sleep. In contrast, the clusters between 31 to 34 Hz (*p* = 0.022), 68 to 70 (*p* = 0.042), 74.5 to 76.5 (*p* = 0.048), 114 to 117 (*p* = 0.027), 118 to 123 Hz (*p* = 0.015), 124 to 151.5 (*p* = 0.001), 155.5 to 164 (*p* = 0.008) and 165 to 170 Hz (*p* = 0.017) were higher in the left hemisphere for the same behavioral state. M1, primary motor cortex; S1, primary somato-sensory cortex; V2, secondary visual cortex; lγ, low gamma or LG; hγ, high gamma or HG; HFO, high frequency oscillations.

During the dark phase, a cluster circumscribed mainly within the theta band in S1-V2 derivation was higher in the right hemisphere during REM sleep. In contrast, clusters of frequencies within LG, HG and HFO were higher in the left hemisphere (Figure 10).

## 3. Discussion

In the present study, we performed a comprehensive analysis of the power and coherence of the iEEG signal of the rat, as well as the impact of behavioral states, cortical areas, laterality (differences between hemispheres) and light/dark phases. iEEG power and coherence were largely affected by behavioral states and recording sites. On the contrary, the influence of the light/dark phases was detected only during sleep. Finally, while we did not find right/left differences in power either in W or sleep, we observed that intra and inter-hemispheric coherence differs between both hemispheres during REM sleep.

### 3.1. Technical Considerations

We performed monopolar recordings (referenced to the cerebellum) utilizing screws (1 mm diameter) in contact with the dura mater as recording electrodes. With this recording design, we observed an important impact of the cortical site. However, as mentioned in the Results Section, the absolute power is highly dependent on the distance between the recording and reference electrodes. In order to discern the relative weight of the power of specific frequency bands in each channel, we also computed the relative power (absolute individual frequencies power normalized by total power). Hence, although complete analyses are provided in the Supplementary Data, in the description of the result, we did not focus on the absolute power as a function of the cortical area or in the total power. In other words, we emphasized the absolute power in the function of behavioral states, and the relative power in the function of the electrode site.

However, it is important to note that total power in OB is lower than in M1 and S1 during NREM sleep (Supplementary Figure S3); this result is not an artifact of the electrode separation (that is the longest in this case), suggesting that the amplitude of the slow waves during NREM sleep in the paleocortex is lower than in the neocortex.

The present as well as most of the studies that analyzed the iEEG spectrum focus on “classical” or “standard” frequency bands, that are associated with behavioral states and cognitive functions [11,19,21,27,28,29]. Nevertheless, the effect of different variables such as moment of the day and laterality on power and coherence could be circumscribed to only a subset of frequencies within a “classical” band or could include changes that extend over these band limits. In these cases, smaller changes could remain undetected when analyzing a whole “classical” frequency band [30]. Because of this, for day/night and laterality analyses, we also performed the result-driven analysis of clusters of frequencies. This methodology allowed us to unveil changes in the iEEG power that not coincide exactly with “standard” or “classical” frequency bands.

### 3.2. iEEG Power

During W, gamma and HFO power reached the maximal level [20,21]; however, although in our analysis we eliminated the epochs with movements artifacts, we cannot rule out the possibility that part of the signal corresponds to muscle activity contamination that reaches cortical electrodes through volume conduction. Interestingly, in the OB, two small deflections in the HG band during W (signaled by blue arrows in Figure 2) are readily observed. Gamma oscillations in the OB have been known since the pioneering study of Adrian (1942) [31]; these oscillations are in phase with the respiratory potentials and significantly increase during active exploration [32,33]. In accordance with this, we found that HG power was higher in the OB during W than during sleep.

NREM sleep was characterized by a higher power spectrum profile, associated with large absolute and relative power values in slow frequency bands (delta, theta and sigma), that can be observed throughout the cortex (Figure 2 and Table 2 and Table 3). A remarkable change in the slope is readily observed at lower frequency bins of the beta band; from this point, there is a marked and constant decrease in power as a function of the frequency. As mentioned in the Introduction, delta (and low theta) is related to the cortical and thalamic slow oscillations, while sigma power is related to sleep spindles; both electrographic features that characterize NREM sleep. As expected, delta and sigma band power during NREM were larger than during W and REM sleep in most of the cortical areas. Furthermore, we found that absolute theta power was higher in NREM than in W and REM sleep in the motor and somatosensory cortices. Hence, even if W and REM sleep exhibit a clear peak at ≈7 Hz in posterior areas, and the relative theta rhythm predominates over other frequency bands, the absolute theta power (considering the whole band) is not as high as in NREM sleep.

During REM sleep, the relative weight of the theta band is highlighted with the analysis of the relative power (Table 2 and Table 3). The main origin of theta rhythm is in the hippocampus; this hippocampal theta rhythm modulates cortical neuronal activity [14,21,34,35]. A prominent peak in theta and relatively large power in gamma and HFO (larger than during NREM sleep) characterize REM sleep [21]. In fact, in the present study, we found that HG power was higher during REM than during NREM sleep in the motor cortex. Additionally, as described before [21], a narrow peak in HFO at ≈130 Hz can be appreciated during REM sleep in the OB and sensory cortices (signaled with red arrows in Figure 2). HFO is implicated in sensory processing [36], and a recent study suggests that the OB is a source of HFO [37]. However, HFO power was not significantly different between NREM and REM sleep, probably because the set of frequencies involved in the peak is much narrower than the whole HFO band.

### 3.3. iEEG Coherence

The spectral coherence is a tool to examine the functional interactions between different cortices as a function of the frequency [19,38]. In accordance with previous studies [19,20,21], during W, large values of intra and inter-hemispheric coherence were observed for high frequencies (HG and HFO) in almost all the derivations. Hence, during W, both gamma and HFO power (that reflects local synchronization) and coherence (that suggest synchronization between areas) are high.

During NREM sleep, delta coherence was higher than during W and REM sleep; thus, large delta power and coherence characterize NREM sleep. However, Pal et al. (2016) [39] found that, during NREM, there was a reduction in the cortico-cortical delta coherence in comparison to W. Of note is that these authors used the mean global coherence (an average of the coherence for the individual channel pairs), and they only evaluated the inter-hemispheric combination of electrodes. It was interesting that delta coherence was larger in homologous inter-hemispheric than in intra-hemispheric derivations (Figure 7); this fact was also demonstrated in humans [22].

Similar to our previous report [21], we found that REM sleep is characterized by large theta coherence, especially between visual and/or somatosensory electrodes. Another valuable issue is that HG and HFO coherence during REM sleep is lower than in W. Similar results have been described in rats [20,39]. Additionally in cats, there are very low gamma coherence values (both LG and HG) during REM sleep [19,27]. In accordance with Cavelli et al. (2018) [21], HFO coherence for intra-hemispheric posterior (S1-V2, sensory) derivations has a clear “peak” during REM sleep (indicated by a black arrow in Figure 6); this peak did not reach statistical significance in comparison to NREM sleep when we analyzed the whole HFO band. In spite of this, it is important to note that HFO intra-hemispheric z’-coherence was significantly higher than inter-hemispheric coherence during REM sleep (Figure 7).

### 3.4. Impact of the Light/Dark Phases

In the present report, we analyzed the iEEG during the subjective day (9 a.m. to 3 p.m.) and compared it with the subjective night (9 p.m. to 3 a.m.); the lights were on from 6 a.m. to 6 p.m. Hence, we evaluated the average of 6 h periods of the light/dark phases in the middle of these phases. In other words, the 3 h at the beginning and at the end of the phases, that should be more unsteady, were not analyzed. This is important to take it into account because previous studies showed an important modification in the hour-to-hour iEEG oscillations [40,41].

Albino rats have short sleep cycles (on average ≈11 min) and are more active during the dark phase; i.e., light phase is their main resting period [42,43,44]. Interestingly, during REM sleep, there was a clear predominance of theta power (≈5–7 Hz) in the light phase (Figure 5); this effect reached the maximum in V2, and it was statistically significant between 4.5 to 7.5 Hz. This predominance during the light phase could be explained by more consolidated REM sleep in the resting phase. In contrast, during NREM sleep, high frequency powers of the iEEG were skewed toward the dark phase (Figure 5). These phenomena could be related to a shallow NREM sleep during the active phase.

To the best of our knowledge, the light/dark phases differences in the iEEG spectral coherence were not studied before. Cluster analysis revealed that there were light/dark phase differences, but only during REM sleep. We found a dark phase predominance within theta, sigma and beta bands in S1 inter-hemispheric derivation (Figure 7). In contrast, there was a light phase predominance for frequencies higher than 173 Hz in S1-V2 intra-hemispheric derivation during this behavioral state (Figure 8). The functional meaning of these day/night differences in electro-cortical coherence confined just to REM sleep is unknown, but may be related to the circadian strength of the cognitive function executed during this behavioral state, such as memory processing [45,46].

### 3.5. Right/Left Hemispheric Differences in Electro-Cortical Activity

There were no differences between the right/left hemispheres’ iEEG power either during light or dark phases during W. Vyazovskiy and Tobler (2008) [24] described iEEG laterality at 4.5–6.0 Hz during a hand preference task; however, as in our results, the authors did not find differences in naïve animals.

During NREM sleep, Vyazovskiy et al. (2002) [41] showed a left-hemispheric predominance of low-frequency power in the parietal cortex at the beginning of the light period, when sleep pressure is high. The left-hemispheric dominance changed to a right-hemispheric dominance in the course of the resting phase when sleep pressure dissipated. Additionally, during recovery from sleep deprivation, parietal left-hemispheric predominance was enhanced. We did not see hemispheric laterality in any frequency band, probably because we did not record the first 3 h of the light phase, which seems to be the time where laterality is mostly developed.

During REM sleep, right-hemispheric predominance in the theta band power was elucidated [41]. Although, no significant differences were observed, in accordance with these authors, there was a clear tendency for right predominance in the theta band power in M1 and S1 during REM sleep, both during light and dark phases (Supplementary Figures S5 and S6).

We did not find previous reports that compared the effect of laterality in the iEEG coherence in rodents; however, changes in iEEG coherence during W have been shown in humans in relation to their skilled hand [47]. In the present report, right/left significant differences in coherence were found only during REM sleep in the dark phase (Figure 10). We found a right predominance of frequencies within the theta range in S1-V2 derivation, and a left predominance for clusters within the gamma and HFO bands. New experimental approaches are needed to explain these differences between both hemispheres.

## 4. Materials and Methods

### 4.1. Experimental Animals

Eleven Wistar male adult rats (270–300 g) were used for this study. The number of animals was selected based on previous studies [21,39,48,49]. The rats were determined to be in good health by veterinarians of the institution. All experimental procedures were conducted in agreement with the National Animal Care Law (#18611) and with the “Guide to the care and use of laboratory animals” (8th edition, National Academy Press, Washington, DC, USA, 2010). Furthermore, the Institutional Animal Care Committee approved the experimental procedures (No 070153-000332-16). Adequate measures were taken to minimize the pain, discomfort or stress of the animals, and all efforts were made to use the minimal number of animals necessary to obtain reliable scientific data. Animals were maintained on a 12-h light/dark cycle (lights on at 6.00 a.m.) and housed five to six per cage before the experimental procedures. Food and water were freely available. Rats were habituated to be cabled to the rotating connector in the sleep chamber for four days before the experiments.

### 4.2. Surgical Procedures

We employed surgical procedures similar to those used in our previous studies [21,50,51]. The animals were chronically implanted with intracranial electrodes. Anesthesia was induced with a mixture of ketamine-xylazine (90 mg/kg; 5 mg/kg i.p., respectively). Rats were positioned in a stereotaxic frame and the skull was exposed. In order to record the iEEG, stainless steel screw electrodes were placed on the skull above the right and left M1, S1 and V2, as well as on the right OB and cerebellum (reference electrode). A representation of the electrode positions and their coordinates, according to [26], are shown in Figure 1A,B. In order to record the electromyogram (EMG), a bipolar electrode was inserted into the neck muscle. The electrodes were soldered into a 12-pin socket and fixed to the skull with acrylic cement. At the end of the surgical procedures, an analgesic (Ketoprofen, 1 mg/kg subcutaneous) was administered. Incision margins were kept clean and a topical antibiotic was applied on a daily basis. After the animals recovered from the preceding surgical procedures, they were adapted to the recording chamber for one week.

### 4.3. Sleep Recordings

Animals were housed individually in transparent cages (40 × 30 × 20 cm) containing wood shaving material in a temperature-controlled (21–24 °C) room, with water and food ad libitum, under a 12:12 h light/dark cycle (lights on at 6 a.m.). Experimental sessions were conducted during the light (9 a.m. to 3 p.m.) and dark periods (9 p.m. to 3 a.m.) in a sound-attenuated chamber that also acts as a Faraday box. The recordings were performed through a rotating connector, to allow the rats to move freely within the recording box. Bioelectric signals were amplified (×1000), filtered (0.1–500 Hz), sampled (1024 Hz, 16 bits) and stored in a PC using Spike 2 software version 9.04 (Cambridge Electronic Design, Cambridge, UK).

### 4.4. Data Analysis

The states of sleep and W were determined in 10 s epochs. W was defined as low voltage fast waves in frontal cortex, a mixed theta rhythm in occipital cortex and relatively high EMG activity. Light sleep (LS) was determined as high voltage slow cortical waves interrupted by low voltage fast iEEG activity. Slow wave sleep (SWS) was defined as continuous high amplitude slow (1–4 Hz) neocortical waves and sleep spindles combined with a reduced EMG activity. LS and SWS were grouped as NREM sleep. REM sleep was defined as low voltage fast frontal waves, a regular theta rhythm in parietal and occipital cortices, and a silent EMG except for occasional twitching. In order to analyze power spectrum (in each channel) and coherence (between pairs of iEEG channels or derivations) we used procedures similar to those done in our previous studies [20,21,50]. The maximum number of non-transitional and artifact-free periods of 30 s was selected during each behavioral state to determine the mean power and coherence for each rat. Power spectrum was estimated by means of the *pwelch* built-in function in MATLAB version R2020a (The MathWorks Inc, Natick, MA, USA) using the following parameters: window = 30 s, noverlap = [], nfft = 2048, fs = 1024, which correspond to employing 30 s sliding windows with half window overlap with a 0.5 Hz resolution. Right M1 was excluded in one rat due to the presence of artifacts.

The coherence between selected pairs of iEEG channels was analyzed in 30 s epochs. We chose for the analysis all the intra-hemispheric and inter-hemispheric pairwise combination of adjacent cortices (the distance between adjacent neocortical electrodes was 5 mm; Figure 1B). For each period, the Magnitude Squared Coherence for each channel (for details about coherence definition see [19,38], was calculated with the *mscohere* built-in MATLAB (parameters: window = 30 s, noverlap, nfft = 2048, fs = 1024). In order to normalize the data and conduct parametric statistical tests, we applied the Fisher z’ transform to the coherence values [19]. The analysis of the data was performed for the classically defined frequency band in rodents: delta, 1–4 Hz; theta, 5–9 Hz; sigma, 10–15 Hz; beta, 16–30 Hz; low gamma (LG), 31–48 Hz; high gamma (HG) 52–95 Hz; high frequency oscillations (HFO), 105–200 Hz [21,50]. Frequencies around 50 and 100 Hz were not analyzed to avoid alternating current artifacts. For the coherence analysis, NREM data were excluded in two rats, and REM data were excluded in one rat, due to artifacts that made processing unfeasible.

Differences in mean power and coherence among states (W, NREM and REM sleep) and electrode position or derivation, were evaluated by means of a two-ways repeated measures mixed-effects model, and Sidak as a correction for multiple comparisons test. We employed a mixed-effects model because we had to remove the information of noisy iEEG channels in 3 rats. We also computed the relative power as the absolute power of a specific frequency band/the sum of the power from 0.5 to 200 Hz. This analysis was performed independently for each state. Statistical significance was set at *p* < 0.05. Partial eta squared (η_p_^2^) was used to evaluate the effect size [52].

In order to determine if power and coherence were different between the time of the day (light vs. dark phases) or between hemispheres (right vs. left), a paired two-tailed Student test was performed for each of the abovementioned bands. As we analyzed seven frequency bands, a Bonferroni correction for multiple comparisons was applied. With this correction *p* < 0.0071 was considered statistically significant. The predominance was calculated by means of the formula: (a − b)/(a + b). “a” represents the mean power for each frequency in the light phase or in the right side, and “b” the mean power in the dark period or in the left side. A positive value means that power during the light period (or right side) was higher than during dark period (or left side) and vice versa.

We are aware that the specific start- and end-points of each frequency band is arbitrary, and vary between subjects [30,53]. Because of this, for the day/night and laterality analyses we also performed a second evaluation by means of a cluster-based permutation test, consisting of comparing empirical clusters of frequencies against a randomized distribution, thus allowing the frequency bands to be delimited in a statistical approach without the need of a previous convention. This method consisted of comparing individual frequencies (512 frequencies) in each condition by means of paired *t*-tests (alpha = 0.05). Once p values were obtained for each frequency, all consecutive significant frequencies were grouped into empirical clusters (defining a minimum cluster size of 4 frequency points), and a new statistic was formed by summing the t-statistic of each frequency inside the cluster. To determine if a given cluster was significant, a null hypothesis distribution of cluster statistics was constructed by randomizing labels (day/night or left/right) and repeating the cluster construction method for a total of 10,000 randomizations. The p values of the empirical clusters were obtained by comparing each cluster statistic to the randomized cluster statistic distribution (X). A one-tailed comparison was employed, where the *p* value was calculated as *p* value = P (X < Xobs) [54].

In summary, as recommended by Simmons et al. (2012), we have reported how we defined our sample size, all data exclusions, all manipulations, and all measures [55].

## 5. Conclusions

In the present study, we carried out a thorough analysis of the spectral power and coherence of the rat iEEG. We found major effects on these parameters in the function of both behavioral states and cortical areas. We also revealed that there are night/day differences in power and coherence during sleep, but not during W. Additionally, while we did not find right/left differences in power either in W or sleep, we observed that, during REM sleep, intra-hemispheric coherence differs between both hemispheres. We consider that this systematic analysis of the iEEG dynamics during physiological W and sleep provides a template or reference for comparison with pharmacological, toxicological or pathological challenges.

## Figures and Tables

**Table 1:** Absolute power.

Frequency	Cortex				Behavioral State				Cortex × State			
	df	*p*	F	η_p_^2^	df	*p*	F	η_p_^2^	df	*p*	F	η_p_^2^
Delta	3, 30	0.0010	7.1	0.415	2, 20	<0.0001	25.0	0.714	6, 57	<0.0001	6.9	0.421
Theta	3, 30	0.0003	8.5	0.459	2, 20	0.0047	7.1	0.415	6, 57	0.0010	4.4	0.316
Sigma	3, 30	0.0003	8.5	0.459	2, 20	<0.0001	24.6	0.711	6, 57	0.0608	2.2	0.188
Beta	3, 30	<0.0001	12.6	0.557	2, 20	0.0488	3.5	0.259	6, 57	0.4312	1.0	0.095
LG	3, 30	0.0066	4.9	0.328	2, 20	0.0017	8.9	0.471	6, 57	0.5688	0.8	0.077
HG	3, 30	0.0001	9.8	0.495	2, 20	<0.0001	59.2	0.855	6, 57	<0.0001	15.5	0.620
HFO	3, 30	0.0002	9.0	0.474	2, 20	<0.0001	57.9	0.852	6, 57	0.0007	4.6	0.326

Statistical evaluation of the absolute spectral power in function of cortical regions, behavioral state, and interaction between both factors. Repeated mixed-effects model. df, degrees of freedom; η_p_^2^ partial eta squared; LG, low gamma; HG, high gamma; HFO, high frequency oscillations.

**Table 2:** Relative power. Repeated measures mixed models

Frequency	Cortex				Behavioral State					Cortex × State		
	df	*p*	F	η_p_^2^	df	*p*	F	η_p_^2^	df	*p*	F	η_p_^2^
Delta	3, 30	0.5710	0.7	0.065	2, 20	<0.0001	41.4	0.805	6, 57	<0.0001	14.9	0.611
Theta	3, 30	<0.0001	35.7	0.781	2, 20	0.0009	10.2	0.504	6, 57	<0.0001	61.2	0.866
Sigma	3, 30	0.0005	8.0	0.444	2, 20	<0.0001	15.9	0.613	6, 57	0.2353	1.4	0.128
Beta	3, 30	0.0389	3.2	0.242	2, 20	0.0084	6.1	0.379	6, 57	0.0166	2.9	0.233
LG	3, 30	0.0544	2.8	0.219	2, 20	<0.0001	26.8	0.728	6, 57	<0.0001	4.4	0.316
HG	3, 30	0.0162	4.0	0.286	2, 20	<0.0001	22.5	0.692	6, 57	0.0213	2.7	0.221
HFO	3, 30	0.0052	5.2	0.342	2, 20	<0.0001	33.6	0.770	6, 57	0.1817	1.5	0.136

Statistical evaluation of the relative spectral power in function of the cortical region, behavioral state, and interaction between both factors. Repeated mixed-effects model.

**Table 3:** Relative power. Sidak multiple comparison test.

State	Comparison	Delta	Theta	Sigma	Beta	LG	HG	HFO
W	OB vs. M1	0.9621	0.8365	0.2279	0.7908	0.0485	0.9294	0.0334
	OB vs. S1	0.6436	0.0452	0.0613	0.0274	0.0494	0.0366	0.0014
	OB vs. V2	0.2194	0.1459	0.6616	0.7521	0.7853	0.0173	0.9786
	M1 vs. S1	0.9897	0.4926	0.9954	0.4594	>0.9999	0.3488	0.9120
	M1 vs. V2	0.7637	0.8360	0.9809	>0.9999	0.5783	0.2254	0.1934
	S1 vs. V2	0.9919	0.9958	0.7595	0.4585	0.5776	>0.9999	0.0137
NREM	OB vs. M1	0.6010	0.9957	0.2926	0.1330	>0.9999	0.9631	0.9925
	OB vs. S1	0.0054	<0.0001	>0.9999	0.9986	>0.9999	0.9711	0.9987
	OB vs. V2	0.8876	0.09998	0.9974	>0.9999	0.9998	0.9986	>0.9999
	M1 vs. S1	0.2591	<0.0001	0.4462	0.3735	>0.9999	>0.9999	>0.9999
	M1 vs. V2	0.9983	0.9957	0.6233	0.0928	0.9998	0.9994	0.9944
	S1 vs. V2	0.0970	<0.0001	0.9999	0.9931	>0.9999	0.9996	0.9991
REM	OB vs. M1	0.9979	0.7428	0.0018	0.0698	0.0872	>0.9999	0.2567
	OB vs. S1	0.5279	<0.0001	0.0318	0.0130	>0.9999	0.1355	0.4773
	OB vs. V2	0.2539	<0.0001	0.9989	0.6463	0.0977	0.0106	0.4965
	M1 vs. S1	0.8384	0.0001	0.9651	0.9854	0.1181	0.0787	0.9999
	M1 vs. V2	0.5548	<0.0001	0.0072	0.8287	<0.0001	0.0051	0.9993
	S1 vs. V2	0.9994	0.3830	0.0930	0.3875	0.1020	0.9561	>0.9999

*p*-values of the Sidak multiple comparisons test, comparing the differences in the relative power between the different cortical region of the right hemisphere. OB, olfactory bulb; M1, primary motor cortex; S1, primary somato-sensory cortex; V2, secondary visual cortex; df, degrees of freedom; η_p_^2^ partial eta squared, W, wakefulness; LG, low gamma; HG, high gamma; HFO, high frequency oscillations.

**Table 4:** Z’-coherence.

Frequency	Derivation				Behavioral State				Derivation × State			
	df	*p*	F	η_p_^2^	df	*p*	F	η_p_^2^	df	*p*	F	η_p_^2^
Delta	5, 50	0.0411	2.5	0.200	2, 20	<0.0001	23.1	0.698	10, 82	0.0121	2.5	0.233
Theta	5, 50	0.0423	2.5	0.200	2, 20	0.0015	9.2	0.479	10, 82	<0.0001	10.7	0.566
Sigma	5, 50	0.1992	1.5	0.130	2, 20	0.0221	4.6	0.315	10, 82	0.0018	3.2	0.280
Beta	5, 50	0.3609	1.86	0.157	2, 20	0.0356	3.9	0.280	10, 82	0.0129	2.4	0.226
LG	5, 50	0.3687	1.1	0.099	2, 20	0.0058	6.7	0.401	10, 82	0.0094	2.6	0.240
HG	5, 50	0.2258	1.4	0.123	2, 20	0.0004	11.7	0.539	10, 82	<0.0001	4.3	0.344
HFO	5, 50	0.0600	2.3	0.187	2, 20	<0.0001	32.1	0.762	10, 82	<0.0001	5.5	0.401

Statistical evaluation of the z’-coherence in function of the derivation, behavioral state, and interaction between both factors. Repeated measures mixed-effects model. df, degrees of freedom; η_p_^2^ partial eta squared; LG, low gamma; HG, high gamma; HFO, high frequency oscillations.

## Supplementary Materials

The following are available online at https://www.mdpi.com/2624-5175/2/4/39/s1, Figure S1: Absolute power in function of behavioral states and cortical regions, Figure S2: Mean absolute power in function of cortical regions, Figure S3: Total power, Figure S4: Differences in relative power in function of behavioral states, Figure S5: Absolute power: right vs. left hemispheric difference during the light phase, Figure S6: Absolute power: right vs. left hemispheric difference during the dark phase, Figure S7: Z’-coherence in function of the derivations, Figure S8: Differences in z’-coherence in function of behavioral states, Figure S9: Z’-coherence in function of the derivation, Figure S10: Intra-hemispheric z’-coherence: right vs. left hemispheric difference during the light phase.

## Abbreviations

EEG	Electroencephalogram
iEEG	Intra-cranial electroencephalogram
HFO	High frequency oscillations
HG	High gamma
LG	Low gamma
M1	Primary motor cortex
NREM	Non-REM
OB	Olfactory bulb
REM	Rapid eye movement
S1	Primary somatosensory cortex
V2	Secondary visual cortex
W	Wakefulness
