# Supplementary material for: Power and Coherence in the EEG of the Rat: Impact of Behavioral States, Cortical Area, Lateralization and Light/Dark Phases
Source: Clocks Sleep. 2020 Dec 9;2(4):536–56. doi: 10.3390/clockssleep2040039 (PMC7768537; doi:10.3390/clockssleep2040039)
Supplement: Supplementary file 1 [file clockssleep-02-00039-s001.pdf]

A

Cortex	Comparison	Delta	Theta	Sigma	Beta	LG	HG	HFO
**OB**	W vs NREM	0.6445	0.4622	0.0123	0.9732	0.0199	<0.0001	<0.0001
	W vs REM	0.1889	0.7319	0.9566	0.4866	0.0957	<0.0001	<0.0001
	NREM vs REM	0.0138	0.0774	0.0031	0.2634	0.9026	0.8339	0.9684
**M1**	W vs NREM	<0.0001	0.0004	<0.0001	0.2875	0.0006	<0.0001	<0.0001
	W vs REM	0.6163	0.6545	>0.9999	0.7885	0.1984	<0.0001	<0.0001
	NREM vs REM	<0.0001	<0.0001	<0.0001	0.0464	0.1231	0.0133	0.3058
**S1**	W vs NREM	<0.0001	0.0064	<0.0001	0.1379	0.0321	<0.0001	<0.0001
	W vs REM	0.8051	0.8555	0.9998	0.9115	0.6745	0.0020	<0.0001
	NREM vs REM	<0.0001	0.0474	<0.0001	0.0331	0.3065	0.0716	0.9649
**V2**	W vs NREM	0.1540	0.8113	0.1495	0.9978	0.1898	0.0023	0.0002
	W vs REM	0.9614	0.7019	0.9994	0.8749	0.1905	0.0451	0.0004
	NREM vs REM	0.0562	0.9973	0.1182	0.9398	>0.9999	0.6695	0.9937

B

States	Comparison	Delta	Theta	Sigma	Beta	LG	HG	HFO
**W**	OB vs M1	0.9741	0.9921	0.9767	0.5754	0.2381	0.0723	0.0001
	OB vs S1	0.8577	0.2735	0.9494	0.2945	0.5623	<0.0001	<0.0001
	OB vs V2	0.2606	0.9897	0.9889	0.7840	>0.9999	<0.0001	<0.0001
	M1 vs S1	0.9995	0.6663	0.9999	0.9981	0.9983	0.0375	0.9171
	M1 vs V2	0.7673	0.7844	0.6850	0.0442	0.1482	<0.0001	0.1486
	S1 vs V2	0.9533	0.0676	0.6048	0.0146	0.4103	0.0016	0.7756
**NREM**	OB vs M1	0.0090	0.0062	0.0124	0.0389	0.9557	0.9998	0.8640
	OB vs S1	<0.0001	0.0010	0.0095	0.0030	0.5119	>0.9999	0.9923
	OB vs V2	0.9203	0.8132	0.4517	0.3883	0.9637	0.8570	0.9568
	M1 vs S1	0.0468	0.9888	>0.9999	0.9344	0.9664	0.9954	0.9981
	M1 vs V2	0.0003	<0.0001	<0.0001	<0.0001	>0.9999	0.9667	>0.9999
	S1 vs V2	<0.0001	<0.0001	<0.0001	<0.0001	0.9591	0.7301	>0.9999
**REM**	OB vs M1	>0.9999	0.9977	0.7833	0.2688	0.1028	0.3289	>0.9999
	OB vs S1	>0.9999	0.0027	0.6559	0.0636	0.0472	0.3550	0.9946
	OB vs V2	0.9997	0.7065	>0.9999	0.9922	>0.9999	0.9576	0.8884
	M1 vs S1	>0.9999	0.0120	>0.9999	0.9844	0.9994	>0.9999	0.9626
	M1 vs V2	0.9978	0.9490	0.5988	0.0685	0.1408	0.0486	0.7387
	S1 vs V2	0.9993	0.1208	0.4684	0.0122	0.0664	0.0581	0.9984

Supplementary Figure 1. Absolute power in function of behavioral states and cortical regions. p values of the Sidak multiple comparisons test, comparing the differences in the absolute power between behavioral states (A) and cortical regions (B). Data show in A are summarized in Figure 3. The data are from the right hemisphere during the light phase. OB, olfactory bulb; M1, primary motor cortex; S1, primary somato-sensory cortex; V2, secondary visual cortex; W, wakefulness; LG, low gamma; HG, high gamma; HFO, high frequency oscillations.

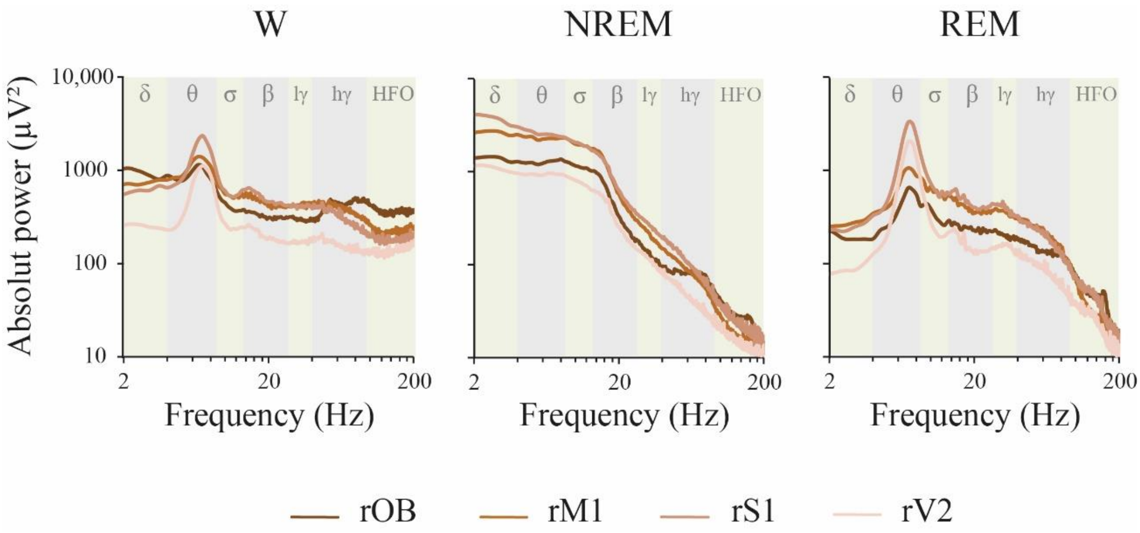

Supplementary Figure 2. Mean absolute power in function of cortical regions. Mean absolute power spectral profile of each behavioral state during the light period for all the electrodes of the right hemisphere. The analyzed frequency bands are indicated by different colors in the background of the graphics. OB, olfactory bulb; M1, primary motor cortex; S1, primary somato-sensory cortex; V2, secondary visual cortex; W, wakefulness; lγ, low gamma or LG; hγ, high gamma or HG; HFO, high frequency oscillations.

Total Power

A

	Localization			Behavioral State			Localization x State		
	Df	p	F	df	p	F	df	P	F
Total power	3,30	0.0001	9.6	2, 20	<0.0001	20.0	6, 57	0.0003	5.2

B

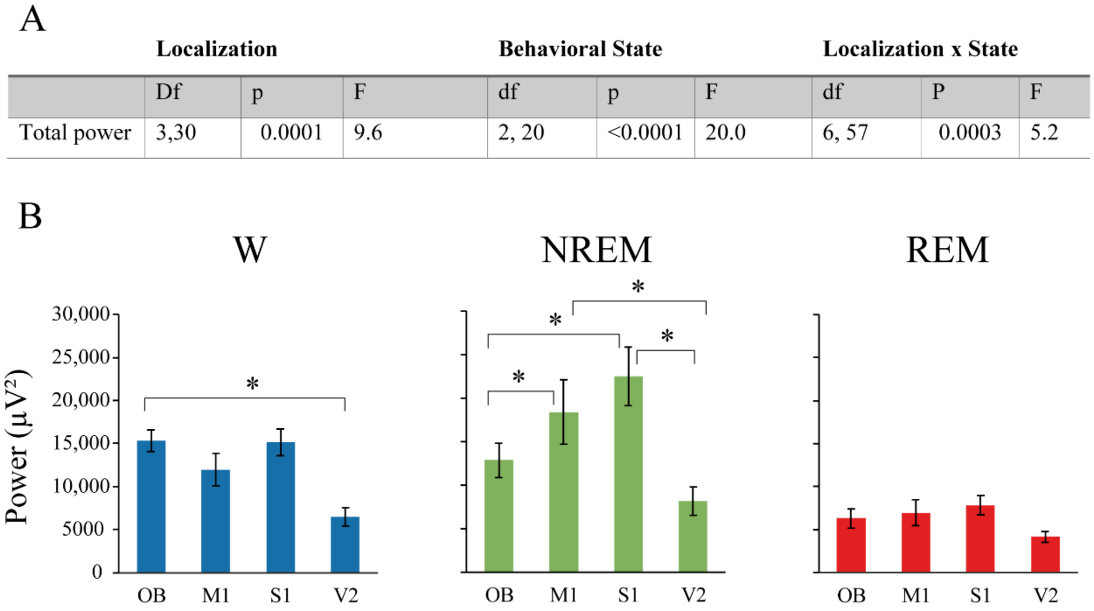

C

State	Comparison	p value
W	OB vs M1	0.4853
	OB vs S1	0.2315
	OB vs V2	0.0005
	M1 vs S1	0.9991
	M1 vs V2	0.1122
	S1 vs V2	0.2784
NREM	OB vs M1	0.0163
	OB vs S1	0.0004
	OB vs V2	0.5393
	M1 vs S1	0.8690
	M1 vs V2	<0.0001
	S1 vs V2	<0.0001
REM	OB vs M1	0.8917
	OB vs S1	0.9284
	OB vs V2	0.9800
	M1 vs S1	>0.9999
	M1 vs V2	0.4337
	S1 vs V2	0.5000

D

Cortex	Comparison	p value
OB	W vs NREM	0.1968
	W vs REM	<0.0001
	NREM vs REM	0.0312
M1	W vs NREM	0.0144
	W vs REM	0.2855
	NREM vs REM	<0.0001
S1	W vs NREM	0.0001
	W vs REM	0.4712
	NREM vs REM	<0.0001
V2	W vs NREM	0.8431
	W vs REM	0.6690
	NREM vs REM	0.2245

Supplementary Figure 3. Total power. A. Statistical evaluation of the total power in function of cortical regions, behavioral state, and interaction between both factors. Repeated mixed-effects model. B. Mean total power in function of cortical regions. Total power is lower in the V2 probably because it is closer to the reference electrode located in the cerebellum. The error bars show the standard error of the mean. Asterisks indicate significant differences, $p < 0.05$. C. p values of the Sidak multiple comparisons test, comparing the differences in the total power between the different cortical localizations of the right hemisphere for each behavioral state. D. p values of the Sidak multiple comparisons test, comparing the differences in the total power between the different behavioral states in each right cortex. OB, olfactory bulb; M1,

primary motor cortex; S1, primary somato-sensory cortex; V2, secondary visual cortex; W, wakefulness.

Cortex	Comparison	Delta	Theta	Sigma	Beta	LG	HG	HFO
**OB**	W vs NREM	0.0032	0.6824	<0.0001	0.0015	0.0314	<0.0001	<0.0001
	W vs REM	0.0926	0.9053	0.1590	0.8238	0.0068	0.1815	<0.0001
	NREM vs REM	<0.0001	0.9710	0.0134	0.0165	<0.0001	<0.0001	0.0964
**M1**	W vs NREM	<0.0001	0.8538	<0.0001	0.0001	<0.0001	<0.0001	<0.0001
	W vs REM	0.2034	0.8360	0.0066	0.2606	0.0172	0.7641	0.0002
	NREM vs REM	<0.0001	>0.9999	0.3633	0.0272	<0.0001	<0.0001	0.6090
**S1**	W vs NREM	<0.0001	<0.0001	0.0195	0.1988	<0.0001	<0.0001	<0.0001
	W vs REM	0.0677	0.0022	0.1235	0.7243	0.8881	0.4177	0.0049
	NREM vs REM	<0.0001	<0.0001	0.8450	0.7589	<0.0001	0.0071	0.5565
**V2**	W vs NREM	<0.0001	0.7643	0.0003	0.0294	0.0028	0.0002	<0.0001
	W vs REM	0.1089	<0.0001	0.5729	0.7624	0.9970	0.1371	<0.0001
	NREM vs REM	<0.0001	<0.0001	0.0115	0.2260	0.0049	0.0861	0.6982

Supplementary Figure 4. Differences in relative power in function of behavioral states. p values of the Sidak multiple comparisons test, comparing the differences in the absolute power in function of behavioral states for each cortical region (of the right hemisphere). OB, olfactory bulb; M1, primary motor cortex; S1, primary somato-sensory cortex; V2, secondary visual cortex; df, degrees of freedom; W, wakefulness; LG, low gamma; HG, high gamma; HFO, high frequency oscillations.

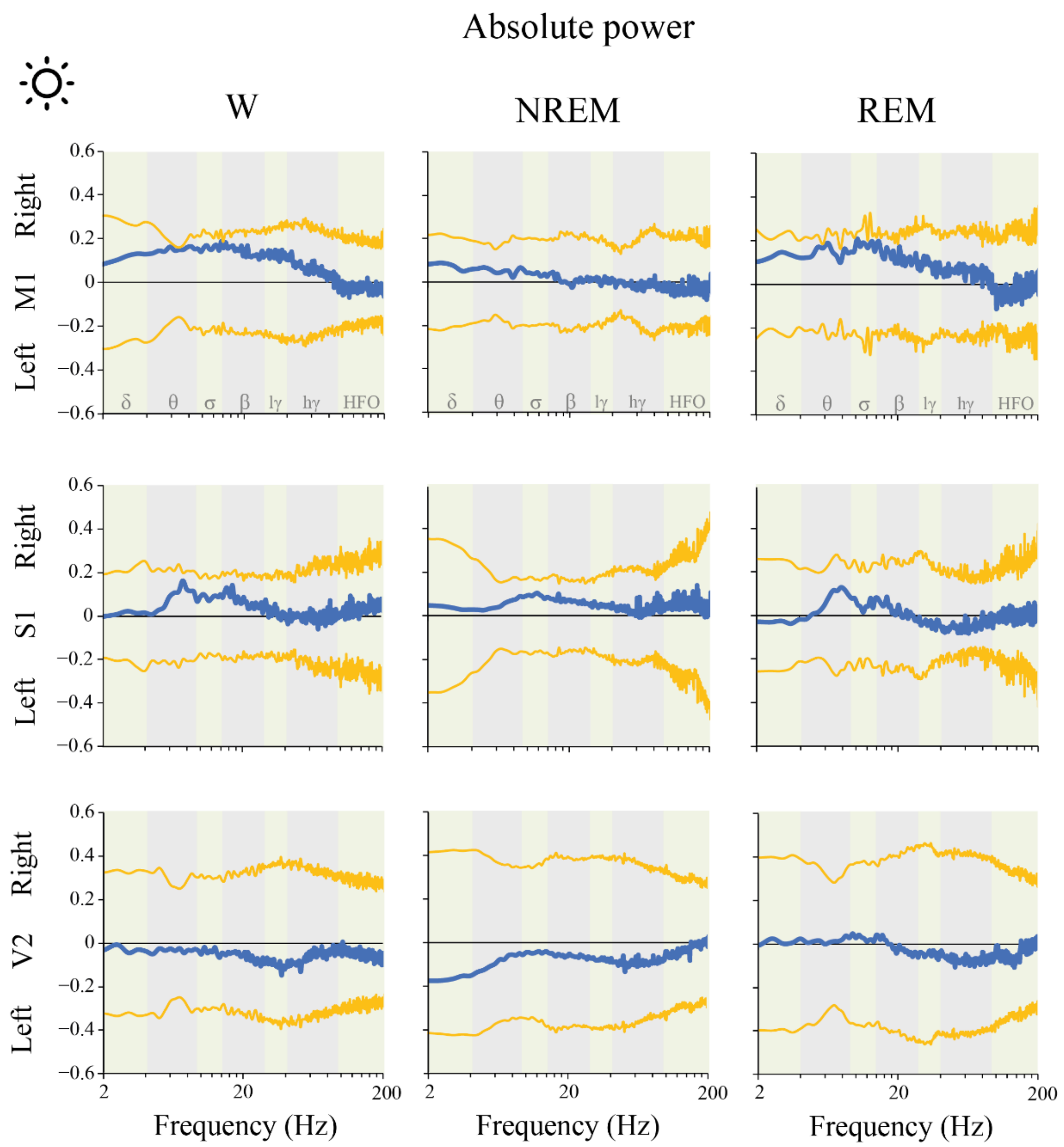

Supplementary Figure 5. Absolute power: right Vs. left hemispheric difference during the light phase. The predominance was calculated by means of the formula: (a-b)/(a+b). "a" represents the mean power for each frequency in the right hemisphere, and "b" the mean power in the left hemisphere. A positive value means that power in the right was higher than in the left hemisphere and *vice versa*. The blue traces indicate the mean power difference between right and dark hemispheres. The yellow lines represent the standard deviation of the mean with respect to zero. The statistical evaluation was performed by the two-tailed paired t-test with Bonferroni correction for multiple comparisons; no significant differences were observed. M1, primary motor cortex; S1, primary somato-sensory cortex; V2, secondary visual cortex; lγ, low gamma or LG; hγ, high gamma or HG; HFO, high frequency oscillations.

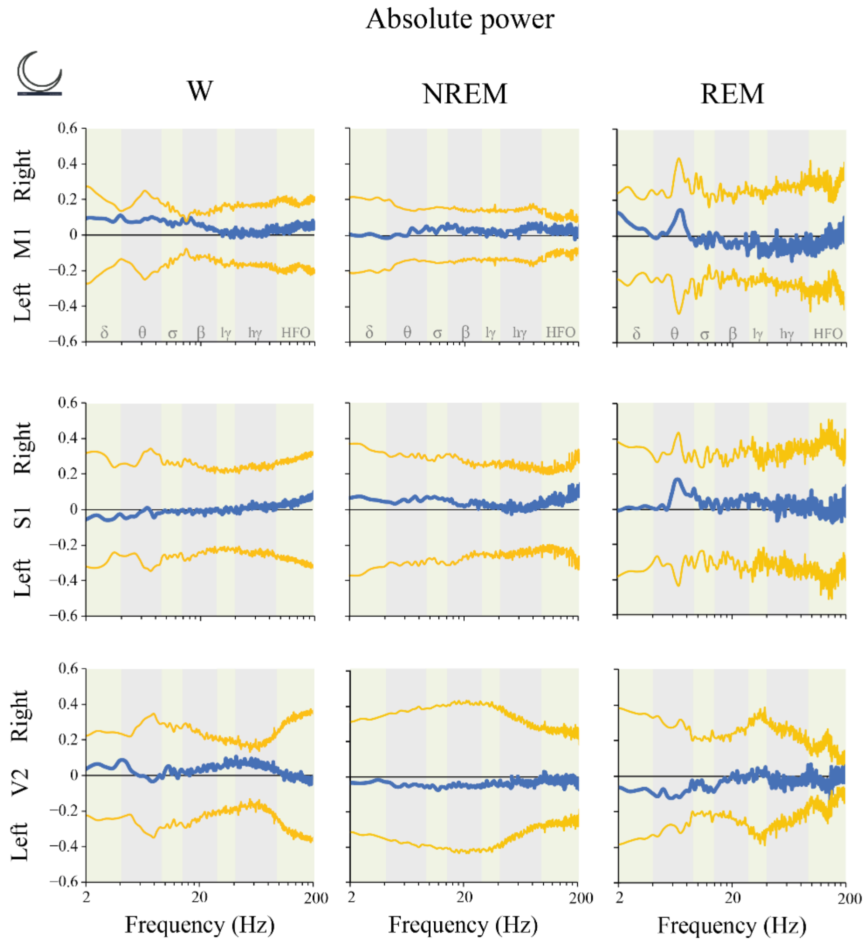

Supplementary Figure 6. Absolute power: right Vs. left hemispheric difference during the dark phase. The predominance was calculated by means of the formula: (a-b)/(a+b). "a" represents the mean power for each frequency in the right hemisphere, and "b" the mean power in the left hemisphere. A positive value means that power in the right was higher than in the left hemisphere and *vice versa*. The blue traces indicate the mean power difference between right and dark hemispheres. The yellow lines represent the standard deviation of the mean with respect to zero. The statistical evaluation was performed by the two-tailed paired t-test with Bonferroni correction for multiple comparisons; no significant differences were observed. M1, primary motor cortex; S1, primary somato-sensory cortex; V2, secondary visual cortex; lγ, low gamma or LG; hγ, high gamma or HG; HFO, high frequency oscillations.

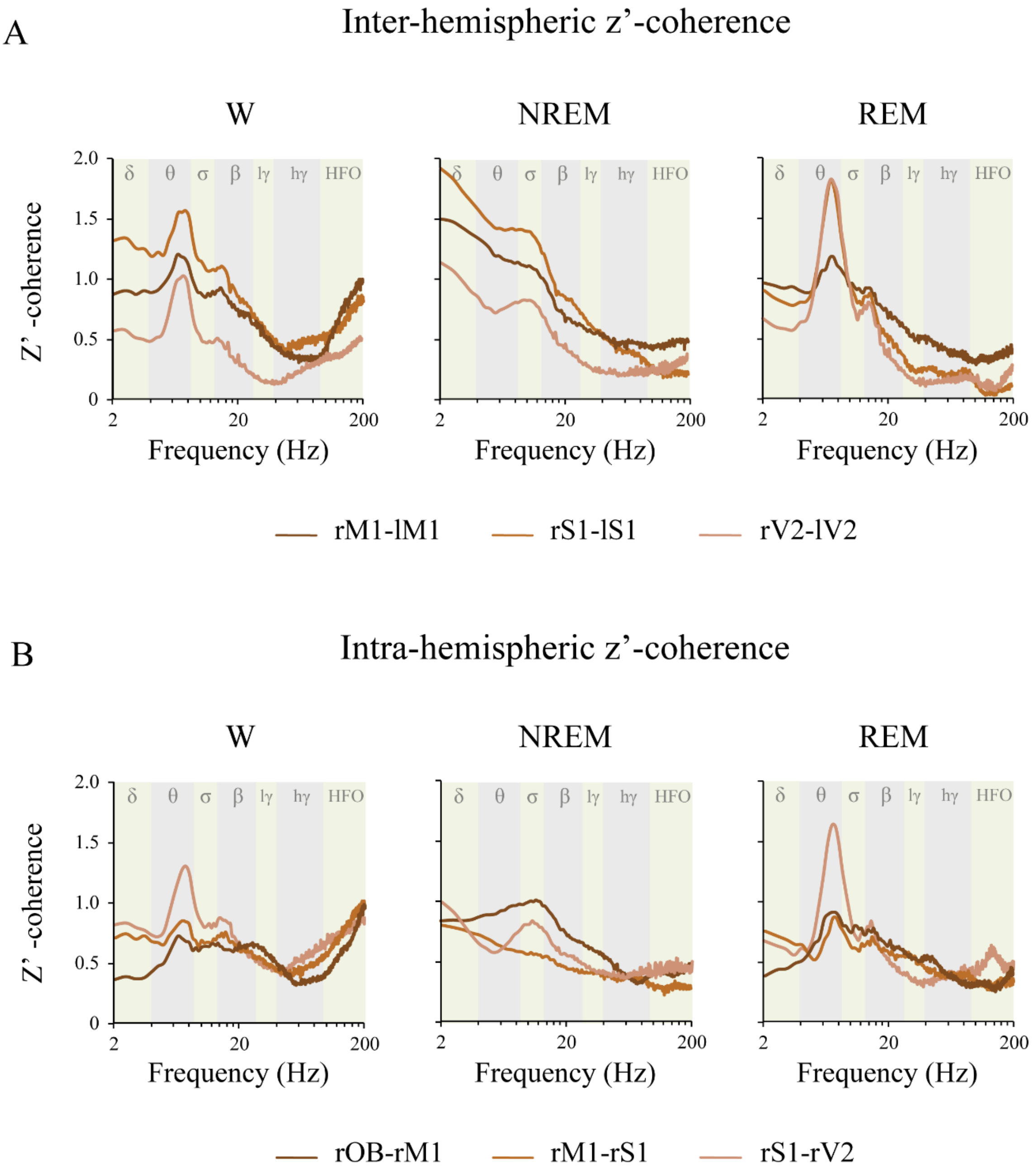

Supplementary Figure 7. Z'-coherence in function of the derivations. Mean z'-coherence profile of the intra-hemispheric (A, for the right hemisphere) and inter-hemispheric (B) derivations during wakefulness (W), NREM and REM sleep in the light phase. The analyzed frequency bands are indicated by different colors in the background of the graphics. OB, olfactory bulb; M1, primary motor cortex; S1, primary somato-sensory cortex; V2, secondary visual cortex; r, right; l, left; lγ, low gamma or LG; hγ, high gamma or HG; HFO, high frequency oscillations.

Derivation		Delta	Theta	Sigma	Beta	LG	HG	HFO
	Intra-hemispheric z'coherence							
rOB – rM1	W vs NREM	0.0014	0.0287	0.0004	0.6053	0.8309	0.9930	0.0007
	W vs REM	0.8569	0.0778	0.1541	0.9999	0.8453	>0.9999	<0.0001
	NREM vs REM	0.0129	0.9520	0.1089	0.6477	>0.9999	0.9935	0.7875
rM1 – rS1	W vs NREM	0.0030	0.9974	0.9635	0.0966	0.8563	0.0898	<0.0001
	W vs REM	>0.9999	0.9664	0.7950	0.6974	0.9471	0.0103	<0.0001
	NREM vs REM	0.0030	0.9192	0.9772	0.9410	0.9929	0.8696	0.9122
rS1 – rV2	W vs NREM	0.9485	<0.0001	0.7063	0.4468	0.9998	0.0021	<0.0001
	W vs REM	0.4460	0.0731	0.9510	0.1566	0.1528	0.0004	<0.0001
	NREM vs REM	0.2151	<0.0001	0.9416	0.9410	0.2083	0.9872	0.9676
	Inter-hemispheric z'coherence							
rM1 – lM1	W vs NREM	<0.0001	0.4673	0.0438	0.9834	0.3244	0.3029	0.0002
	W vs REM	0.9196	0.9992	0.9847	0.6268	0.9990	0.8330	<0.0001
	NREM vs REM	<0.0001	0.3982	0.0937	0.8552	0.2631	0.0648	0.3631
rS1 – lS1	W vs NREM	0.0004	0.6795	0.8996	0.0922	0.4033	<0.0001	<0.0001
	W vs REM	0.5178	0.0446	0.7298	0.0011	0.0034	<0.0001	<0.0001
	NREM vs REM	<0.0001	0.0031	0.3474	0.4359	0.2082	>0.9999	0.6549
rV2 – lV2	W vs NREM	0.0002	0.6415	0.0062	0.0283	0.0011	>0.9999	0.4611
	W vs REM	0.9918	<0.0001	0.0019	0.7857	0.9989	0.0322	0.0006
	NREM vs REM	0.0005	<0.0001	0.9944	0.1954	0.0007	0.0479	0.0567

Supplementary Figure 8. Differences in z'-coherence in function of behavioral states. p values of the Sidak multiple comparisons test, comparing the differences in the z'-coherence in function of behavioral states for each frequency band. OB, olfactory bulb; M1, primary motor cortex; S1, primary somato-sensory cortex; V2, secondary visual cortex; r, right; l, left; W, wakefulness; LG, low gamma; HG, high gamma; HFO, high frequency oscillations.

A

Comparison	Delta	Theta	Sigma	Beta	LG	HG	HFO
rOB-rM1 vs rM1-rS1	0.9955	>0.9999	>0.9999	>0.9999	>0.9999	>0.9999	0.9988
rOB-rM1 vs rM1-rV2	0.1567	0.0751	0.5571	0.9847	0.9947	0.2005	0.4356
rOB-rM1 vs rM1-lM1	0.8031	0.8266	0.9969	>0.9999	>0.9999	>0.9999	>0.9999
rOB-rM1 vs rS1-lS1	0.0235	0.0461	0.3920	0.9867	>0.9999	0.9520	0.9995
rOB-rM1 vs rV2-lV2	>0.9999	0.9985	>0.9999	0.9967	0.8911	>0.9999	0.7529
rM1-rS1 vs rS1-rV2	0.8703	0.2318	0,7258	0.9695	0.9755	0.6631	0.9533
rM1-rS1 vs rM1-lM1	>0.9999	0.9846	0.9998	>0.9999	>0.9999	>0.9999	>0.9999
rM1-rS1 vs rS1-lS1	0.3692	0.1547	0.5566	0.9730	>0.9999	>0.9999	>0.9999
rM1-rS1 vs rV2-lV2	>0.9999	>0.9999	>0.9999	0.9830	0.9592	0.9946	0.1782
rS1-rV2 vs rM1-lM1	0.9986	0.9755	0.9981	0.9999	0.9926	0.2924	0.7587
rS1-rV2 vs rS1-lS1	>0.9999	>0.9999	>0.9999	>0.9999	>0.9999	0.9890	0.9727
rS1-rV2 vs rV2-lV2	0.5609	0.5958	0.2650	0.2091	0.1661	0.0673	0.0027
rM1-lM1 vs rS1-lS1	0.8428	0.9313	0.9860	0.9999	>0.9999	0.9847	>0.9999
rM1-lM1 vs rV2-lV2	0.9960	>0.9999	0.9340	0.7698	0.9079	>0.9999	0.4025
rS1-lS1 vs rV2-lV2	0.1430	0.4580	0.9963	0.2179	0.5124	0.7255	0.1447

B

Comparison	Delta	Theta	Sigma	Beta	LG	HG	HFO
rOB-rM1 vs rM1-rS1	0.9998	>0.9999	>0.9999	>0.9999	>0.9999	>0.9999	>0.9999
rOB-rM1 vs rM1-rV2	0.6713	0.0777	0.9631	0.9998	>0.9999	0.8373	0.3460
rOB-rM1 vs rM1-lM1	0.8441	0.9997	>0.9999	>0.9999	>0.9999	>0.9999	>0.9999
rOB-rM1 vs rS1-lS1	0.1835	0.0395	0.9393	>0.9999	>0.9999	>0.9999	0.9976
rOB-rM1 vs rV2-lV2	>0.9999	0.4186	>0.9999	0.9891	0.8162	0.9974	0.9729
rM1-rS1 vs rS1-rV2	0.9951	0.0203	0.6591	0.9928	0.9966	0.8247	0.1439
rM1-rS1 vs rM1-lM1	0.9997	0.9701	0.9921	>0.9999	>0.9999	>0.9999	>0.9999
rM1-rS1 vs rS1-lS1	0.7377	0.0094	0.5897	>0.9999	>0.9999	>0.9999	>0.9999
rM1-rS1 vs rV2-lV2	>0.9999	0.1581	>0.9999	0.9995	0.9721	0.9979	0.9993
rS1-rV2 vs rM1-lM1	>0.9999	0.5115	>0.9999	>0.9999	0.9997	0.8352	0.2841
rS1-rV2 vs rS1-lS1	>0.9999	>0.9999	>0.9999	>0.9999	0.9999	0.9349	0.0257
rS1-rV2 vs rV2-lV2	0.9442	>0.9999	0.9692	0.5786	0.3192	0.1553	0.0104
rM1-lM1 vs rS1-lS1	0.9986	0.3355	0.9997	>0.9999	>0.9999	>0.9999	0.9992
rM1-lM1 vs rV2-lV2	0.9895	0.9614	>0.9999	0.9549	0.9088	0.9975	0.9867
rS1-lS1 vs rV2-lV2	0.4753	0.9985	0.9481	0.8407	0.8832	0.9835	>0.9999

C

Comparison	Delta	Theta	Sigma	Beta	LG	HG	HFO
rOB-rM1 vs rM1-rS1	0.9985	>0.9999	0.9963	>0.9999	>0.9999	>0.9999	>0.9999
rOB-rM1 vs rM1-rV2	0.9362	>0.9999	>0.9999	>0.9999	0.9988	0.8319	0.8575
rOB-rM1 vs rM1-lM1	0.2233	0.9882	>0.9999	>0.9999	>0.9999	>0.9999	>0.9999
rOB-rM1 vs rS1-lS1	0.0182	0.7371	0.9798	>0.9999	>0.9999	>0.9999	0.9994
rOB-rM1 vs rV2-lV2	0.9997	>0.9999	>0.9999	0.9975	0.9960	>0.9999	>0.9999
rM1-rS1 vs rS1-rV2	>0.9999	0.9996	0.8436	0.9275	0.9538	0.8801	0.4688
rM1-rS1 vs rM1-lM1	0.8948	0.8642	0.8392	0.9995	>0.9999	>0.9999	0.9999
rM1-rS1 vs rS1-lS1	0.2544	0.4073	0.3459	0.9755	>0.9999	>0.9999	>0.9999
rM1-rS1 vs rV2-lV2	>0.9999	>0.9999	>0.9999	>0.9999	>0.9999	>0.9999	>0.9999
rS1-rV2 vs rM1-lM1	0.9954	>0.9999	>0.9999	>0.9999	0.9996	0.9895	0.9579
rS1-rV2 vs rS1-lS1	0.5884	0.9619	>0.9999	>0.9999	0.9999	0.9125	0.2274
rS1-rV2 vs rV2-lV2	>0.9999	0.9999	0.9834	0.7859	0.5706	0.4580	0.5166
rM1-lM1 vs rS1-lS1	0.9992	>0.9999	>0.9999	>0.999	>0.9999	>0.9999	0.9917
rM1-lM1 vs rV2-lV2	0.8305	0.9025	0.9824	0.9910	0.9906	0.9982	>0.9999
rS1-lS1 vs rV2-lV2	0.1946	0.4658	0.6645	0.8924	0.9828	>0.9999	>0.9999

Supplementary Figure 9. Z'-coherence in function of the derivation. p values of the Sidak multiple comparisons test, comparing the differences in the z'-coherence according to the derivation, during wakefulness (A), NREM (B) and REM sleep (C). OB, olfactory bulb; M1, primary motor cortex; S1, primary somato-sensory cortex; V2, secondary visual cortex; LG, low gamma; HG, high gamma; HFO, high frequency oscillations.

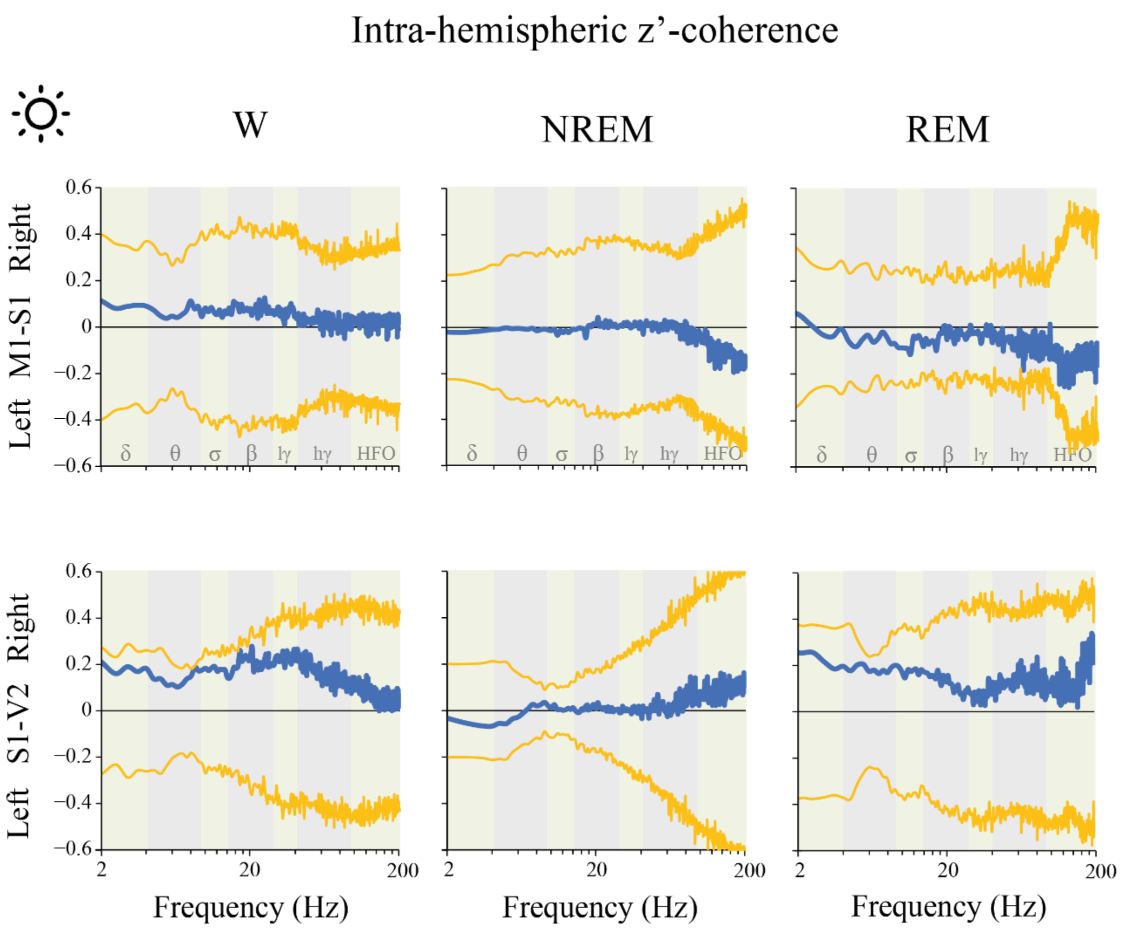

Supplementary Figure 10. Intra-hemispheric z’-coherence: right Vs. left hemispheric difference during the light phase. The predominance was calculated by means of the formula: (a-b)/(a+b). “a” represents the mean z’-coherence for each frequency in the light phase, and “b” the mean coherence during the dark period. A positive value means that z’-coherence in the light period was higher than during dark period and *vice versa*. The blue traces indicate the mean z’-coherence difference between light and dark phases. The yellow lines represent the standard deviation of the mean with respect to zero. The statistical evaluation was performed by the two-tailed paired t-test with Bonferroni correction for multiple comparisons; no significant differences were observed. M1, primary motor cortex; S1, primary somato-sensory cortex; V2, secondary visual cortex; lγ, low gamma or LG; hγ, high gamma or HG; HFO, high frequency oscillations.
